# Prevalence and factors associated with the perception of perineal laceration: a cross-sectional study with data from the Nascer no Brasil Survey, 2011 and 2012

**DOI:** 10.1590/S2237-96222024V33E2023621.EN

**Published:** 2024-04-05

**Authors:** Luciana Mamede, Daniele Marano, Marcos Augusto Bastos Dias, Paulo Roberto Borges de Souza

**Affiliations:** 1 Fundação Oswaldo Cruz, Instituto Nacional de Saúde da Mulher, da Criança e do Adolescente Fernandes Figueira. Programa de Pós Graduação em Saúde da Mulher e da Criança. Rio de Janeiro, RJ, Brazil; 2 Fundação Oswaldo Cruz, Instituto Nacional de Saúde da Mulher, da Criança e do Adolescente Fernandes Figueira, Rio de Janeiro, RJ, Brazil; 3 Fundação Oswaldo Cruz, Instituto de Informação e Comunicação Científica e Tecnológica da Fundação Oswaldo Cruz, Rio de Janeiro, RJ, Brazil

**Keywords:** Perineum, Rupture, Spontaneous, Natural Childbirth, Pelvic Floor Disorders, Maternal Health, Cross-sectional Studies, Perineo, Ruptura Espontánea, Parto Normal, Trastornos del Suelo Pél-vico, Salud Maternal, Estudios Transversales, Períneo, Ruptura Espontânea, Parto Normal, Distúrbios do Assoalho Pélvico, Saúde Materna, Estudos Transversais

## Abstract

**Objective:**

To describe the prevalence of perineal laceration, based on the self-reported perception of postpartum women, and to analyze factors associated with its occurrence in Brazil.

**Methods:**

This was a cross-sectional study conducted with 23,894 postpartum women, excluding twin pregnancies, cesarean sections, and births with episiotomies, between 2011 and 2012. Prevalence ratios (PR) and 95% confidence intervals (95%CI) of association between the event and maternal, fetus/newborn, obstetric and clinical management characteristics were estimated in hierarchical Poisson regression models.

**Results:**

Out of 4,606 postpartum women, 49.5% (95%CI 46.1;42.9) self-reported perineal laceration. Being an adolescent (PR = 1.12; 95%CI 1.02;1.25), primipara (PR = 1.47; 95%CI 1.33;1.63), having had excessive gestational weight gain (PR = 1.17; 95%CI 1.07;1.29) and having undergone the Kristeller maneuver (PR = 1.18; 95%CI 1.08;1.29) increased the proportion of the outcome.

**Conclusion:**

The results found call for prenatal care and adjustments to childbirth care so as to be in accordance with current recommendations.

## Study contributions

**Main results:**

Prevalence of self-reported perineal laceration was 49.5%. Being in the adolescent age group, primiparity, excessive gestational weight and the Kristeller maneuver were risk factors associated with the event.

**Implications for services:**

Studying self-reported prevalence of perineal laceration supports new care practices, highlights the prevention of risk factors considered modifiable and confirms the need to follow current guidelines.

**Perspectives:**

New national studies are needed comparing prevalence of self-reported perineal laceration with that recorded in medical records in order to support care practices and public obstetric policies.

## Resumo

**Objetivo:**

Descrever a prevalência da laceração perineal segundo a percepção autorrelatada da puérpera, e analisar os fatores associados à sua ocorrência no Brasil.

**Métodos:**

Estudo transversal conduzido em 23.894 puérperas, excluindo-se gestações gemelares, cesarianas e partos com episiotomias entre 2011 e 2012. Razões de prevalência (RP) e intervalos de confiança de 95% (IC_95%_) da associação entre o evento e as características maternas, feto/recém-nato, obstétricas e manejo clínico foram estimadas em modelos de regressão de Poisson hierarquizados.

**Resultados:**

Entre 4.606 puérperas, 49,5% (IC_95%_ 46,1;42,9) autorrelataram laceração perineal. Ser adolescente (RP = 1,12; IC_95%_ 1,02;1,25), primípara (RP = 1,47; IC_95%_ 1,33;1,63), ter tido ganho de peso gestacional excessivo (RP = 1,17; IC_95%_ 1,07;1,29) e ter sido submetida à manobra de Kristeller (RP = 1,18; IC_95%_ 1,08;1,29) elevaram a proporção do desfecho.

**Conclusão:**

Os resultados encontrados demandam atenção pré-natal e adequações na assistência ao parto conforme recomendações vigentes.

## Resumen

**Objetivo:**

Describir la prevalencia de laceración perineal, a partir de la percepción autoinformada de puérperas, y analizar los factores asociados a su aparición en Brasil.

**Métodos:**

Estudio transversal entre 2011 y 2012, con 23.894 puérperas, excluyendo embarazos gemelares, cesáreas y partos con episiotomías. Se estimaron razones de prevalencia (RP) e intervalos de confianza del 95% (IC_95%_) de la asociación entre el evento y las características maternas, feto/recién nacido, obstétricas y de manejo clínico en modelos de regresión jerárquica de Poisson.

**Resultados:**

Entre 4.606 mujeres en posparto, el 49,5%(IC_95%:_46,1;42,9) informó laceración perineal. Ser adolescente (RP = 1,12; IC_95%_ 1,02;1,25), primipara (RP = 1,47; IC_95%_ 1,33;1,63), haber tenido aumento excesivo de peso gestacional (RP = 1,17; IC_95%_ 1,07;1,29) y haber sido sometido a la maniobra de Kristeller (RP = 1,18; IC_95%_ 1,08;1,29) aumentó la proporción de resultados.

**Conclusión:**

Los resultados encontrados requieren atención prenatal y ajustes en la atención del parto de acuerdo con las recomendaciones actuales.

## INTRODUCTION

In 2018, the World Health Organization (WHO) published the document entitled “WHO recommendations: Intrapartum care for a positive childbirth experience”, contemplating the prevention and reduce perineal trauma, this being relevant in the intrapartum period.^1^

Perineal trauma is an injury that occurs in the perineum during vaginal delivery, which can compromise other anatomical structures of the pelvic floor. It can be produced by perineal laceration, representing spontaneous rupture of the tissue during the passage of the cephalic pole of the newborn, and/or by episiotomy, a surgical incision performed by the professional.^2^ The American College of Obstetricians and Gynecologists (ACOG) considers that the expected frequency inherent to some degree of laceration in vaginal delivery varies from 53% to 73%.^2^

Perineal lacerations are classified into degree I, II, III or IV, and although degree III and IV are less prevalent, they can further compromise the tissue plane, structures and functions of the pelvic floor. They can trigger sexual disorders (chronic pain and dyspareunia), gynecological disorders (discomfort during gynecological examination), urinary disorders (urinary incontinence), as well as coloproctological and pelvic organ prolapse disorders, requiring multidisciplinary therapeutic care.^3^ ^),(^ ^4^

Perineal lacerations are multifactorial, highlighting their association with maternal characteristics (age between 27 and 30 years, primiparity and gestational age ≥ 42 weeks), characteristics related to the fetus/newborn (head circumference > 35 cm),^4^ obstetric characteristics and those related to the procedures and interventions of the obstetric team during labor (second stage of labor lasting more than two hours, instrumental vaginal birth, use of oxytocin and induced labor).^1^ ^),(^ ^4^ In relation to degrees III and IV, the ACOG considers the main factors associated with the outcome to be the newborn’s weight above 4 kg, shoulder dystocia, type of fetal occiput posterior position, women of Asian ethnicity, primiparity, prolonged expulsion stage and instrumental delivery associated or not with episiotomy.^2^

In the literature, data on the prevalence of perineal laceration are divergent,^2^ in addition we did not find articles that jointly analyze associated factors and prevalence in the Brazilian context. Therefore, this study aimed to describe the prevalence of perineal laceration based on the self-reported perception of postpartum women and to analyze factors associated with its occurrence in Brazil.

## METHODS

This was a cross-sectional study using data from the national survey entitled “Nascer no Brasil: Inquérito Nacional sobre Parto e Nascimento” (Born in Brazil: National survey on Childbirth and Birth, coordinated by the Oswaldo Cruz Foundation (FIOCRUZ), conducted between between 2011 and 2012, and organized into three stages. In the first stage, 266 hospitals were selected, being public, private or a mixture of both, with 500 or more births/year in the Brazilian macro-regions, both in state capitals and interior regions. In the second stage, seven days were calculated to obtain a sample of 90 postpartum women per hospital. Finally, in the third stage, postpartum women were selected until the final sample of 23,894 postpartum women was obtained. Other details about the survey can be found in the study by Leal et al.^5^ Eligible women were those who gave birth to a live newborn or stillborn weighing ≥ 500 g and/or gestational age ≥ 22 weeks, excluding those with severe mental disorder, deaf or foreigners who did not understand the Portuguese language.^5^

The exclusion criteria used by this study were having had a cesarean section, having twins, having a medical record showing performance of episiotomy, self-reporting performance or suspected performance of episiotomy and/or being Asian or Indigenous. The data we analyzed were obtained from the women’s medical records, questionnaires and/or prenatal cards. The dependent variable was the women’s self-reported perception of perineal laceration, obtained by means of the following question as part of the questionnaire administered with the women: *Do you know what your perineum (vagina) was like after giving birth?* The answer options were as follows: it wasn’t torn, it wasn’t cut and there were no stitches; it tore a little, but didn’t need stitches; I didn’t get stitches, but I don’t know if it was torn; it was torn and I had stitches; they cut and stitched it; I had stitches, but I don’t know if it tore or if the doctor cut it; unable to inform. Women who replied “it tore a little, but didn’t need stitches” and “it was tore and I had stitches” were classified as having perineal laceration according to their perception. The remaining women were classified as not having perineal laceration according to their perception, and the reply “I had stitches, but I don’t know if it tore or if the doctor cut it” was not considered to indicate laceration because of the possibility of including women who underwent episiotomy, as this was an exclusion criteria in this study.

**Figure 1:**
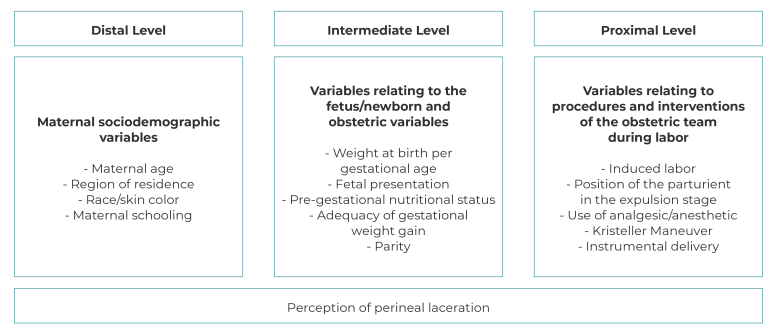
‒ Theoretical conceptual framework of predictive factors of perineal laceration

We considered the following independent variables: maternal sociodemographic characteristics, fetus/newborn characteristics, obstetric characteristics and the procedures and interventions of the obstetric team during labor, organized into distal, intermediate and proximal hierarchical levels (Figure 1).

On the distal level, maternal sociodemographic aspects obtained from the postpartum women’s questionnaire were included: maternal age range in years (12-19; ≥ 20-34; ≥ 35); Brazilian region of residence (North; Northeast; Southeast; South; Midwest); race/skin color (White; Black; mixed race; Asian; Indigenous); schooling (incomplete elementary education; complete elementary education; incomplete high school education; complete high school education; complete higher education and above). In view of the small sample size regarding Asian and Indigenous race/skin color, we decided to exclude this group from the analyses.

On the intermediate level, birth weight for gestational age was classified based on the International Fetal and Newborn Growth Consortium for the 21^st^ Century (INTERGROWTH-21^st^), whereby newborns were classified as small for gestational age (below the 10^th^ percentile), appropriate for gestational age, between the 10^th^ and 90^th^ percentile), and large for gestational age (above the 90^th^ percentile).^6^ ^)^ Fetal presentation was categorized as cephalic, breech or shoulder.^5^ ^)^ These data were collected from medical records.

With regard to obstetric characteristics, we considered pre-gestational nutritional status (low weight: < 18.5 kg/m^2^; normal weight: ≥ 18.5 to ≤ 24.9 kg/m^2^; overweight: ≥ 25 to ≤ 29.9 kg/m^2^; obese: ≥ 30 kg/m^2^),^7^ ^)^ adequacy of gestational weight gain (insufficient; adequate; excessive) and parity (primipara; multipara). In order to calculate adequacy of gestational weight gain, we took weight at the end of pregnancy or weight at the last medical consultation, less pre-gestational weight, both self-reported or collected from the prenatal card, whereby adequacy was corrected for gestational age.^5^

On the proximal level, we considered variables related to obstetric team management and intervention during labor: induced labor (no; yes); position of the parturient in the expulsion stage (vertical; horizontal); analgesic/anesthetic use (no; yes); Kristeller maneuver (no; yes); instrumental delivery (forceps/vacuum cup: no; yes). These data were collected from medical records.

The variables were organized in a theoretical conceptual framework by level of proximity to the outcome, including predictive factors for perineal laceration based on criteria defined by the ACOG and Tavares et al.^2^ ^),(^ ^4^

The data were input to the Fundação Oswaldo Cruz (Fiocruz) REDCap application and analyzed using Stata 13 software. Prevalence ratios were estimated by adjusting Poisson regression models. In addition to the analysis model for each study covariate, the covariates of the three levels that were significant (p-value < 0.05) were kept in the final adjusted model. The analyses were performed using the Stata survey module because data were obtained through complex sampling.

The main study was approved by the Fiocruz Escola Nacional de Saúde Pública (ENSP) Research Ethics Committee (Opinion No. 92/10; Certificate of Submission for Ethical Appraisal [Certificado de Apresentação para Apreciação Ética - CAAE]: No. 0096.0.031.000-10), in keeping with the free and informed consent form. Our study was submitted to the Instituto Nacional de Saúde da Mulher, da Criança e do Adolescente Fernandes Figueira Research Ethics Committee and approved as per Opinion No. 5.486.223/CAAE: 57347922.2.0000.5269, in accordance with National Health Council Resolution No. 466/2012.

## RESULTS

**Figure 2:**
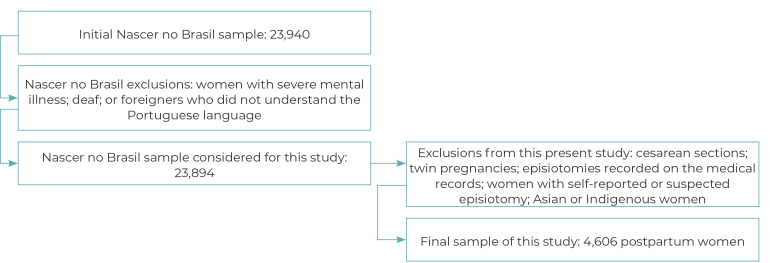
‒ Exclusion and sample flowchart

Of the total number of women who participated in the larger study (23,894), 19,288 postpartum women were excluded for the following reasons: 12,409 women who had cesarean sections, 43 with twin pregnancies, 5,683 with a record of episiotomy in their medical records, 1,063 with self-reported or suspected episiotomy and 90 of Asian or Indigenous race/skin color (Figure 2), so that our study consisted of 4,606 women who had recently given birth vaginally, 49.5% (95%CI 46.1;42.9) of whom self-reported perineal laceration.

Of the postpartum women evaluated, 71.5% were aged between 20 and 34 years, 39.7% lived in the Southeast region of Brazil, 64.3% were of mixed race/skin color, 40.3% had incomplete elementary education.

Regarding the characteristics of the fetus, 99.0% had cephalic presentation and 79.8% were classified as appropriate for gestational age. Regarding obstetric characteristics, 73% were multiparous, 34.7% had excessive gestational weight gain. Regarding obstetric team procedures and interventions, 96.4% of the postpartum women did not receive analgesia/anesthesia and 24.3% underwent the Kristeller maneuver (Table 1).

**Table 1:** ‒ Maternal sociodemographic profile, relating to the fetus/newborn, obstetrics and procedures and interventions of the obstetric team during labor, Brazil, 2011-2012

Variables	n^a^	%
Maternal age (years)		
12-19	831	19.0
20-34	3,315	71.5
35 or over	459	9.5
Region of residence		
North	743	12.8
Northeast	1,538	33.3
Southeast	1,325	39.7
South	681	9.7
Midwest	319	4.6
Race/skin color		
White	1,112	24.7
Black	478	11.0
Mixed race	3,014	64.3
Maternal schooling		
Incomplete elementary education	1,967	40.3
Complete elementary/incomplete high school education	1,265	29.2
Complete high school education	1,268	28.6
Complete higher education and above	106	1.9
Weight at birth per gestational age		
Small for gestational age	367	7.9
Appropriate for gestational age	3,606	79.8
Large for gestational age	619	12.3
Fetal presentation		
Cephalic	4,121	99.0
Breech	39	1.1
Adequacy of gestational weight gain		
Insufficient	1,538	32.6
Adequate	1,490	32.7
Excessive	1,578	34.7
Pre-gestational nutritional status		
Low weight	428	9.1
Normal weight	2,780	61.2
Overweight	1,028	22.1
Obesity	370	7.6
Parity		
Primipara	1,108	27.1
Multipara	3,498	73,0
Induced labor		
No	3,989	85.4
Yes	617	14.6
Position of the parturient during the expulsion period		
Horizontal	4,782	89.3
Vertical	575	10.7
Use of analgesic/anesthetic		
No	4,374	96.4
Yes	163	3.6
Kristeller Maneuver		
No	3,411	75.7
Yes	1,195	24.3
Instrumental delivery		
No	4,551	99.0
Yes	55	1.0

a) n: Unweighted sample size.

In the bivariate analysis, the following distal level variables were associated with the outcome: maternal age between 12 and 19 years (PR = 1.27; 95%CI 1.12;1.43), living in the Northern region (PR = 0.59; 95%CI 0.45;0.76), Southern region (PR = 0.84; 95%CI 0.73;0.97), Northeast region (PR = 0.76; 95%CI 0.66;0.88), Black race/skin color (PR = 0.81; 95%CI 0.70;0.95), mixed race/skin color (PR = 0.83; 95%CI 0.76;0.90), incomplete elementary school education (PR = 0.56; 95%CI 0.47;0.69) and complete elementary school education (PR = 0.78; 95%CI 0.65;0.93). On the intermediate level, the variables associated with laceration were: having had a baby that was small for gestational age (PR = 0.75; 95%CI 0.61;0.92), breech fetal presentation (PR = 0.40; 95%CI 0.17;0.90), having had insufficient gestational weight gain (PR = 0.89; 95%CI 0.80;0.99) and being primipara (PR = 3.21; 95%CI 2 .40;4.30). On the proximal level, the following were associated: induced labor (PR = 1.13; 95%CI 1.02;1.25), use of analgesia (PR = 1.30; 95%CI 1.07;1.58), Kristeller maneuver (PR = 1.22; 95%CI 1.10;1.36) and use of forceps/vacuum cup (PR = 0.62; 95%CI 0.52;0.75) (Table 2).

**Table 2:** ‒ Bivariate analysis of maternal sociodemographic factors, relating to the fetus/newborn, obstetrics and procedures and interventions of the obstetric team during labor, Brazil, 2011-2012

Variables	Perineal laceration			
	No	Yes	PR^a^	95%CI^b^
	n (%)	n (%)		
Distal level				
Maternal age (years)				
12-19	407 (15.0)	615 (23.3)	1.27	1.12;1.43
20-34	2,009 (74.0)	1,815 (68.7)	1.00	‒^c^
35 or over	298 (11.0)	210 (8.0)	0.87	0.75;1.01
Region of residence				
North	456 (16.8)	236 (8.9)	0.59	0.45;0.76
Northeast	968 (36.3)	791 (30.0)	0.76	0.66;0.88
Southeast	897 (33.0)	1,226 (48.5)	1.00	‒^c^
South	268 (9.9)	255 (9.7)	0.84	0.73;0.97
Midwest	111 (4.1)	131 (5.0)	0.93	0.78;1.10
Race/skin color				
White	563 (20.7)	734 (27.8)	1.00	‒^c^
Black	311 (11.4)	268 (10.2)	0.81	0.70;0.95
Mixed race	1,784 (65.7)	1,598 (60.6)	0.83	0.76;0.90
Maternal schooling				
Incomplete elementary education	1,341 (49.3)	821 (31.1)	0.56	0.47;0.69
Complete elementary/incomplete high school education	750 (27.6)	817 (30.9)	0.78	0.65;0.93
Complete high school education	592 (21.8)	933 (35.3)	0.91	0.76;1.09
Complete higher education and above	35 (1.3)	69 (2.6)	1.00	‒^c^
Intermediate level				
Weight at birth per gestational age				
Small for gestational age	258 (9.5)	160 (6.1)	0.75	0.61;0.92
Appropriate for gestational age	2,112 (78.0)	2,159 (81.8)	1.00	‒^c^
Large for gestational age	337 (12.4	320 (12.1)	0.96	0.83;1.11
Fetal presentation				
Cephalic	2,430 (98.3)	2,409 (99.6)	1.00	‒^c^
Breech	42 (1.7)	10 (0.4)	0.40	0.17;0.90
Pre-gestational nutritional status				
Low weight	230 (8.5)	257 (9.7)	1.03	0.89;1.20
Normal weight	1,640 (60.3)	1,646 (62.4)	1.00	‒^c^
Overweight	630 (23.2)	543 (20.6)	0.91	0.82;1.01
Obesity	219 (8.0)	194 (7.3)	0.91	0.78;1.07
Adequacy of gestational weight gain				
Insufficient	965 (35.5)	776 (29.4)	0.89	0.80;0.99
Adequate	890 (32.7)	867 (32.8)	1.00	‒^c^
Excessive	863 (31.7)	997 (37.8)	1.20	1.08;1.33
Parity				
Primipara	448 (16.5)	1,005 (38.1)	3.21	2.40;4.30
Multipara	2,270 (83.5)	1,635 (61.9)	1.00	‒^c^
Proximal level				
Induced labor				
No	2,362 (86.9)	2,214 (83.9)	1.00	‒^c^
Yes	355 (13.1)	426 (16.1)	1.13	1.02;1.25
Position of the parturient in the expulsion stage				
Horizontal	2,427 (89.3)	2,355 (89.2)	1.00	‒^c^
Vertical	290 (10.7)	285 (10.8)	1.10	0.71;1.71
Use of analgesic/anesthetic				
No	2,604 (97.3)	2,494 (95.3)	1.00	‒^c^
Yes	72 (2.7)	122 (4.7)	1.30	1.07;1.58
Kristeller Maneuver				
No	2,168 (79.8)	1,891 (71.6)	1.00	‒^c^
Yes	550 (20.2)	748 (28.4)	1.22	1.10;1.36
Instrumental delivery forceps/vacuum cup				
No	2,705 (99.5)	2,598 (98.4)	1.00	‒^c^
Yes	12 (0.5)	41 (1.6)	0.62	0.52;075

a) PR: Prevalence ratio; b) 95%CI: 95% confidence interval; c) feature (‒): Information not applicable.

In the multivariate analysis, the followed raised the proportion of the outcome: maternal age between 12 and 19 years (PR = 1.12; 95%CI 1.02;1.25), primiparity (PR = 1.47; 95%CI 1.33;1.63), excessive gestational weight gain (PR = 1.17; 95%CI 1.07;1.29) and Kristeller maneuver (PR = 1.18; 95%CI 1.08;1.29). While living in the Northeast region (PR = 0.84; 95%CI 0.74;0.95) and Northern region (PR = 0.65; 95%CI 0.52;0.81), having incomplete elementary education (PR = 0.67; 95%CI 0.55;0.83), having had a baby that was small for gestational age (PR = 0.77; 95%CI 0.64;0.93) and having gained insufficient gestational weight (PR = 0.88; 95%CI 0.81;0.96) reduced the proportion of the outcome (Table 3).

**Table 3:** ‒ Multivariate analysis of maternal sociodemographic factors, relating to the fetus/newborn, obstetrics and procedures and interventions of the obstetric team during labor, Brazil 2011-2012

	Model 1 Distal variables		Model 2 Distal and intermediate variables		Model 3 Distal, intermediate and proximal variables	
Variables	PR^a^	95%CI^b^	PR^a^	95%CI^b^	PR^a^	95%CI^b^
Maternal age (years)						
12-19	1.36	1.22;1.51	1.14	1.02;1.27	1.12	1.02;1.25
20-34	1.00	‒	1.00	‒	1.00	‒
35 or over	0.91	0.79;1.04	0.98	0.84;1.14	1.00	0.86;1.16
Race/skin color						
White	1.00	‒	1.00	‒	1.00	‒
Black	0.90	0.78;1.03	0.89	0.78;1.01	0.89	0.78;1.01
Mixed race	0.94	0.86;1.02	0.93	0.84;1.03	0.93	0.85;1.03
Region of residence						
North	0.64	0.51;0.81	0.67	0.54;0.83	0.67	0.52;0.81
Northeast	0.86	0.75;0.98	0.87	0.77;0.99	0.87	0.74;0.95
Southeast	1.00	‒	1.00	‒	1.00	‒
South	0.87	0.77;0.99	0.89	0.78;1.02	0.90	0.78;1.03
Midwest	0.96	0.82;1.12	1.03	0.87;1.21	0.99	0.85;1.16
Maternal schooling						
Incomplete elementary education	0.57	0.47;0.70	0.68	0.56;0.84	0.67	0.55;0.83
Complete elementary education	0.73	0.61;0.88	0.86	0.71;1.05	0.89	0.73;1.09
Complete high school education	0.88	0.73;1.07	0.96	0.80;1.16	1.00	0.82;1.22
Complete higher education and above	1.00	‒	1.00	‒	1.00	‒
Weight at birth per gestational age						
Small for gestational age			0.76	0.62;0.93	0.77	0.64;0.93
Appropriate for gestational age	‒	‒	1.00	‒	1.00	‒
Large for gestational age			1.13	0.97;1.30	1.11	0.96;1.28
Fetal presentation						
Cephalic	‒	‒	1.00	‒	1.00	‒
Breech			0.50	0.23;1.09	0.50	0.21;1.20
Adequacy of gestational weight gain						
Insufficient			0.89	0.81;0.97	0.88	0.81;0.96
Adequate	‒	‒	1.00	‒	1.00	‒
Excessive			1.18	1.07;1.31	1.17	1.07;1.29
Parity						
Primipara			1.50	1.36;1.65	1.47	1.33;1.63
Multipara	‒	‒	1.00	‒	1.00	‒
Induced labor						
No	‒	‒	‒	‒	1.00	‒
Yes					0.94	0.85;1.04
Parturient position during the expulsion period						
Horizontal	‒	‒	‒	‒	1.00	‒
Vertical					1.02	0.86;1.21
Use of analgesic and anesthetic						
No	‒	‒	‒	‒	1.00	‒
Yes					1.02	0.86;1.22
Kristeller Maneuver						
No	‒	‒	‒	‒	1.00	‒
Yes					1.18	1.08;1.30
Instrumental delivery forceps/vacuum cup						
No	‒	‒	‒	‒	1.00	‒
Yes					1.14	0,93;1,39

a) PR: Prevalence ratio; b) 95%CI: 95% confidence interval.

## DISCUSSION

Being an adolescent or primipara woman, having had excessive gestational weight gain and having undergone a Kristeller maneuver during childbirth were associated with self-reported perception of perineal laceration. While living in the North or Northeast region, having incomplete elementary education, having a baby that was small for gestational age and having insufficient gestational weight gain, reduced the proportion of the outcome.

Although the prevalence of perineal laceration found in this study was different from that reported in other studies,^8^ ^),(^ ^9^ it was similar to that estimated by the ACOG.^2^

In their analysis of the medical records of a cohort of 935 parturient women who did not undergo episiotomy and gave birth at a university hospital in Belo Horizonte, Minas Gerais, between 2013 and 2014, Monteiro et al. found that 78.2% of those women suffered some degree of perineal laceration.^9^ Likewise, a prospective analysis of 222 vaginal births without episiotomy in the city of Recife, Pernambuco, between 2012 and 2013, found that the prevalence of perineal laceration was 79.7%, whereby the authors suggested that the high occurrence of the outcome could be associated with interventional care of health teams, even in a humanized context.^7^ ^)^ On the other hand, a cross-sectional study carried out on 3,255 parturient women in Iran,^8^ in 2015, found a prevalence of laceration (16%) lower than that found in the aforementioned Brazilian studies and in ACOG studies.^2^ The authors highlighted professional training as a measure for improving women’s care and quality of life.^8^

With regard to the distal level (maternal sociodemographic factors), maternal age, region of residence and maternal schooling were associated with the outcome.

The analysis showed that the proportion of perineal laceration was 14% higher among adolescents (12 and 19 years old) compared to adult women. These results are in keeping with the results of a retrospective study carried out in Romania with 1,498 parturient women, of whom 298 were adolescents, between 2020 and 2021, which found 89% higher odds of laceration among adolescents when compared to adults.^10^ ^)^ Greater frequency of perineal laceration during adolescence can be attributed to the immaturity of pelvic bone growth and muscle development, leading to a reduction in the internal diameters of the pelvis and the muscular strength of the pelvic floor, increasing the risk of adverse birth outcomes, such as perineal laceration.^11^

We found that living in the North and Northeast regions of Brazil was associated with lower prevalence of laceration based on the perception of the postpartum women. The United Nations Children’s Fund (UNICEF), in partnership with the Network for the Humanization of Child Delivery and Birth (Rede pela Humanização do Parto e Nascimento), evaluated perinatal care in Brazil, based on Ministry of Health data on deliveries carried out between 2000 and 2017, and found inequalities in the availability and quality of obstetric care nationwide.^12^ Based on this survey, UNICEF concluded that although there was an increase in access to prenatal care in primary care and that Rede Cegonha network had been implemented during this period, there was less prenatal care available in the North and Northeast regions of Brazil, suggesting the inadequate organization of obstetric care associated with the poorer living conditions in those regions.^12^ ^),(^ ^13^

However, the results we found, regarding the lower prevalence of the event among postpartum women living in the North and Northeast regions and among those with a lower level of education, must be evaluated with caution, since these factors can influence the perception of care and health status of this population.

Although obstetric interventions associated with pain, fear, tension and anxiety may favor the occurrence of adverse birth outcomes, these outcomes may be less perceived in vulnerable contexts.^14^ A meta-analysis carried out in Ethiopia in 2018, which included 20 studies and involved a sample of 13,744 pregnant women, estimated the effect of maternal schooling on knowledge and preparation for childbirth and decision-making capacity in the face of obstetric complications, and noted that pregnant women with higher levels of education had greater knowledge and preparation for the childbirth process.^14^

According to the WHO 2018 Recommendations, a higher level of maternal schooling can promote better understanding and adherence to prenatal and labor guidelines.^1^

Among the intermediate level variables (fetus/newborn characteristics and obstetric characteristics), adequacy of birth weight in relation to gestational age, maternal gestational weight gain and parity were associated with the occurrence of perceived perineal laceration.

According to the literature, macrosomia (newborn weight ≥ 4,000 g) increases the probability of dystocic births and may require instrumental delivery, increasing the risk of perineal laceration.^15^ In our study, delivery of newborns who were small for gestational age was a protective factor regarding perception of perineal laceration. These results agree with data from a study conducted in Sweden with 212,101 newborns, between 2006 and 2015, which found lower odds of serious perineal lacerations among mothers of those newborns.^16^ As such, lower biometric measurements (weight and length) partially justify these results, as they reduce mechanical challenges in relation to the path of the birth canal and perineum.^15^ ^)-(^ ^17^

Still in relation to the intermediate level variables, a higher proportion of the outcome was observed among primiparous women. These results are in keeping with the meta-analysis conducted by Wilson & Homer,^18^ involving 12 studies published between 2013 and 2018, and a sample of 515,161 postpartum women, which showed 3.2 times higher odds of laceration among primiparous women when compared to multiparous women.^18^ Confirming these results, cross-sectional study with data from medical records of 421 postpartum women with vaginal birth in Ceará, between 2016 and 2018, it found a higher percentage of lacerations among primiparous women (53.4%) when compared to multiparous women (46.6%).^19^ Greater risk of perineal laceration among primiparous women has been explained by the parturient’s lack of knowledge about the functionality and self-control of the perivaginal muscles during childbirth, associated with the fear-anxiety-pain cycle, leading to excessive activation of muscles that have not yet experienced elasticity and distensibility arising when there have been previous births.^17^

In addition to these factors, some interventions carried out by healthcare teams, such as restricting mobility and keeping the parturient in a lithotomy position, may favor the occurrence of perineal lacerations in primiparous women.^18^

Although it is not possible to avoid primiparity, it is possible to guide procedures, as per the WHO 2018 Recommendations, such as performing perineal massage from the 35^th^ prenatal week onwards, in order to prevent more serious lacerations.^1^

A systematic review with meta-analysis of 50 clinical trials, involving 17,221 pregnant women, showed that perineal massage was effective in preserving the perineum, reducing the risk of perineal laceration.^20^ It also highlighted that the influence of educational activities during prenatal care can inform pregnant women and encourage them to adopt different positions during labor, reducing the risk of interventions and perineal trauma.^20^

Regarding maternal characteristics, insufficient gestational weight gain was a protective factor for the outcome, while excessive weight gain increased the prevalence of the outcome. Maternal gestational weight can be predictive of biometric changes in the fetus/newborn, which can result in dystocic births, predisposing to perineal laceration.^15^ It is noteworthy that excessive gestational weight gain predisposes to macrosomia and consequently to perineal laceration.^21^

A retrospective study of 4,127 births that occurred in Milan, Italy, between 2016 and 2020, found that postpartum women with excess gestational weight gain and mothers of large for gestational age newborns had 2.04 times greater odds of severe perineal lacerations when compared to women with insufficient weight gain and small for gestational age newborns, recommending control of gestational weight in order to reduce adverse birth outcomes.^21^

With regard to the proximal level variables, relating to health team procedures and interventions in childbirth care, the Kristeller maneuver was the only variable statistically associated with the outcome. A study with similar results, carried out in Peru by Becerra-Chauca & Failoc-Rojas, in 2016, found that among 116 births with Kristeller maneuver intervention, perineal lacerations occurred in 32.8% of them.^22^ The maneuver promotes intense downward pressure towards the pelvic floor, favoring association with instrumental delivery and, consequently, perineal laceration.^1^

It should be highlighted there have been WHO recommendations to eliminate this maneuver and similar interventions since 1996,^1^ and this recommendation was incorporated into Brazilian Ministry of Health guidelines in Brazil in 2017. The Kristeller maneuver was proscribed in the WHO global birth guidelines in 2018.^1^ ^),(^ ^23^ Notwithstanding, in the Brazilian birth guidelines published by the Ministry of Health in 2022, there is no mention of support or ratification of the current WHO proscription.^24^

In our study, data relating to perineal laceration extracted from medical records showed a high degree of incompleteness, with this event being recorded in only 25% of the parturient women studied, this being a much lower prevalence rate than that obtained from self-reporting. According to Correa et al.,^25^ ^)^ data from medical records need to have sufficient completeness, quality and reliability in order to adequately represent the reality studied, contributing to the planning of health care strategies and related policies.^25^

A systematic review and meta-analysis conducted by the WHO, with 74 review studies published between 2004 and 2016, involving 300,000 vaginal births, occurring in 41 countries, examined laceration rates in low - and middle-income countries, including Brazil.^26^ According to that study, rates of perineal trauma, indicators of health care quality, were above 70% in low- and middle-income countries and suggested underreporting. The study concluded that there is heterogeneity in care and professional practices and notifications, as well as difficulties in collecting this data from birth records, resulting in a lack of knowledge of the true parameters.^26^

It is also noteworthy that the majority of articles do not evaluate women’s perception of interventions during childbirth. Health service user perception allows evaluation of public health policies and provides support for their improvement.^20^ In the 2016 Cochrane meta-analysis review, covering 20 selected studies, with data from 15,181 women with perineal laceration, only one study evaluated their perception.

There are gaps regarding women’s opinions and perceptions regarding perineal trauma, beyond the simple investigation of satisfaction with birth outcomes.^27^ Roper et al. (2020)^28^ ^)^ evaluated and compared 13 guidelines on perineal trauma and their recommendations, published between 2008 and 2019, using the validated instrument called Appraisal of Guidelines for Research and Evaluation (AGREE) II, and suggested that the formulation of guidelines should include interested parties, such as those affected by the problem, taking their opinions into consideration as relevant elements in the process.^28^

Therefore, although self-reporting does not constitute an accurate source for measuring the outcome, especially in areas with lower socioeconomic and educational levels, the high level of incompleteness and underreporting of the event in medical records, found in this study, guided the decision to use self-reported perception of parturient women as a source of information. Information on other factors that could influence the estimate of the event, such as previous perineal trauma, dystocia, head circumference, was not collected in the main study and is another limitation of our study.

Although the data used in this study were collected more than a decade ago, between 2011 and 2012, they are national in scope and included women cared for in public and private services, serving as a basis for comparative analyses on obstetric care in future studies, such as the 2022-2023 Nascer no Brasil Survey.

Based on the results found, the need to adopt recommendations and practices aimed at preventing perineal laceration in routine prenatal care and childbirth is confirmed. Actions aimed at training, attitudes and improving health professional practices and procedures and promoting interdisciplinary assistance in humanized maternal care are needed.

## References

[1] World Health Organization (WHO) (2018). Positive childbirth experience.

[2] American College of Obstetricians and Gynecologists (ACOG) (2018). Committee on Practice Bulletins-Obstetrics. ACOG Practice Bulletin No. 198: Prevention and management of obstetric lacerations at vaginal delivery. Obstet Gynecol.

[3] Camargo JCS, Varela V, Ferreira FM, Chofakian CBN, Osava RH, Araújo NM (2019). Perineal outcomes and its associated variables of water births versus non-water births: a cross-sectional study. Rev Bras Saude Mater Infant.

[4] Tavares NVS, Dantas NPM, Cardoso ACG, Sanches METL, Araújo ST, Moura RS (2022). Factors that influence the occurrence of perineal laceration in birth. RSD.

[5] Leal MC, Silva AAM, Dias MAB, Gama SGN, Rattner D, Moreira ME (2012). Birth in Brazil: national survey into labour and birth. Reprod Health.

[6] Villar J, Ismail LC, Victora CG, Ohuma EO, Bertino E, Altman DG (2014). International standards for newborn weight, length, and head circumference by gestational age and sex; the Newborn Cross-Sectional Study for the INTERGROWTH-21st Project. Lancet.

[7] Lins VML, Katz L, Vasconcelos FBL, Coutinho I, Amorim MM (2019). Factors associated with spontaneous perineal lacerations in deliveries without episiotomy in a university maternity hospital in the city of Recife, Brazil: a cohort study. J Matern Fetal Neonatal Med.

[8] Abedzadeh-Kalahroudi M, Talebian A, Sadat Z, Mesdaghinia E (2019). Perineal trauma: incidence and its risk factors. J Obstet Gynaecol.

[9] Monteiro MVC, Pereira GM, Aguiar RA, Azevedo RL, Correia-Junior MD, Reis ZS (2016). Risk factors for severe obstetric perineal lacerations. Int Urogynecol J.

[10] Matei A, Poenaru E, Dimitriu MCT, Zaharia C, Ionescu CA, Navolan D (2021). Obstetrical soft tissue trauma during spontaneous vaginal birth in the Romanian Adolescent Population-Multicentric Comparative Study with Adult Population. Int J Environ Res Public Health.

[11] Ronayne ET (2020). Immature pelvic growth and obesity: a biocultural analsysis of risks associated with adolescent pregnancy in the United States. The Arbutus Review.

[12] Rattner D (2021). Assistência ao parto e nascimento: uma agenda para o século 21.

[13] Nunes AD da S, Amador AE, Dantas AP de QM, Azevedo UN de, Barbosa IR (2017). Acesso à assistência pré-natal no Brasil: análise dos dados da Pesquisa Nacional de Saúde. Rev Bras Promoc Saúde.

[14] Ketema DB, Leshargie CT, Kibret GD, Assemie MA, Petrucka P, Alebel A (2020). Effects of maternal education on birth preparedness and complication readiness among Ethiopian pregnant women: a systematic review and meta-analysis. BMC Pregnancy Childbirth.

[15] Turkmen S, Johansson S, Dahmoun M (2018). Foetal macrosomia and foetal-maternal outcomes at birth. J Pregnancy.

[16] Vieira MC, Relph S, Persson M, Seed PT, Pasupathy D (2019). Determination of birth-weight centile thresholds associated with adverse perinatal outcomes using population, customised, and Intergrowthcharts: a Swedish population-based cohort study. Plos Medicine.

[17] Baracho E (2018). Fisioterapia aplicada à saúde da mulher.

[18] Wilson AN, Homer CSE (2020). Third- and fourth-degree tears: A review of the current evidence for prevention and management. Aust N ZJ Obstet Gynaecol.

[19] Sousa LS, Souto REM, Fernandes BKC, Esteche CMGCE, Damasceno AKC, Melo LPT (2021). Maternal indicators of assisted deliveries in a normal in-hospital delivery center. Rev Enferm Atual In Derme.

[20] Silva ML, Santos ABPS, Leite LWC, Silva CEC, Oliveira AN, Teixeira AA (2023). The effectiveness of interventions in the prevention of perineal trauma in parturients: a systematic review with meta-analysis. Eur J Obstet Gynecol Reprod Biol.

[21] Ornaghi S, Fumagalli S, Galimberti S, Ornago AM, Brivio V, Lambicchi L (2023). Adverse childbirth and perinatal outcomes among healthy, low-risk pregnant women with abnormal total gestational weight gain. J Womens Health.

[22] Becerra-Chauca N, Failoc-Rojas VE (2019). Maniobra Kristeller, consecuencias físicas y éticas según sus protagonistas. Rev Cubana Obstet Ginecol.

[23] Brasil. Ministério da Saúde. Secretaria de Ciência, Tecnologia e Insumos Estratégicos (2017). Diretrizes nacionais de assistência ao parto normal: versão resumida. Departamento de Gestão e Incorporação de Tecnologias em Saúde.

[24] Brasil. Ministério da Saúde (2022). Diretriz Nacional de Assistência ao Parto Normal. Versão preliminar.

[25] Correia LOS, Padilha BM, Vasconcelos SML (2014). Methods for assessing the completeness of data in health information systems in Brazil: a systematic review. Cienc Saude Colet.

[26] Aguiar M, Farley A, Hope L, Amin A, Shah P, Manaseki-Holland S (2019). Birth-related perineal trauma in low- and middle-income countries: a systematic review and meta-analysis. Matern Child Health J.

[27] Aasheim V, Nilsen ABV, Reinar LM, Lukasse M (2017). Perineal techniques during the second stage of labour for reducing perineal trauma. Cochrane Database Syst Rev.

[28] Roper JC, Amber N, Wan OYK, Sultan AH, Thakar R (2020). Review of available national guidelines for obstetric anal sphincter injury. Int Urogynecol J.

